# The role of cell replacement in benthic–pelagic coupling by suspension feeders

**DOI:** 10.1098/rsos.160484

**Published:** 2016-11-30

**Authors:** Amanda S. Kahn, Sally P. Leys

**Affiliations:** Department of Biological Sciences, University of Alberta, Edmonton, Alberta, CanadaT6G 2E9

**Keywords:** Porifera, stem cells, cell turnover, sponge loop, choanocytes

## Abstract

Benthic–pelagic coupling through suspension feeders and their detrital pathways is integral to carbon transport in oceans. In food-poor ecosystems however, a novel mechanism of carbon recycling has been proposed that involves direct uptake of dissolved carbon by suspension feeders followed by shedding of cells as particulate carbon. We studied cell replacement rates in a range of cold-water sponge species to determine how universal this mechanism might be. We show that cell replacement rates of feeding epithelia in explants vary from 30 hours up to 7 days, and change during different seasons and life-history stages. We also found that feeding epithelia are not replaced through direct replication but instead arise from a population of stem cells that differentiate and integrate into epithelial tissues. Our results reveal a surprising amount of complexity in the control of cell processes in sponges, with cell turnover depending on environmental conditions and using stem cells as rate-limiting mechanisms. Our results also suggest that for species in cold water with high particulate organic matter, cell turnover is not the mechanism delivering carbon flux to surrounding communities.

## 1. Introduction

Suspension feeders transfer carbon to the benthos by capturing and concentrating plankton from the water column [1], but exactly how carbon is transferred is still not clear. Traditional work has focused on detrital pathways [1] which include direct fallout of particulate organic material (POM), filtration of POM and excretion of wastes, and use of dissolved organic matter (DOM) mediated by microbes (microbial loop).

A fourth pathway has come to light that involves uptake of DOM by animals directly (such as sponges) and sloughing of tissues [2]. The latter has been termed the ‘sponge loop’ and in fact may be so substantial that it could provide all of Darwin's missing carbon in oligotrophic coral reefs [2]. This idea has resonated with a wide range of fields from ecology [3,4] to natural products and drug discovery [5], evolutionary developmental biology [6,7], aquarist publications [8] and cancer research [9], but it has not yet been demonstrated how broadly applicable this mechanism is. Cell shedding by sponges is a very different carbon transfer mechanism from the detrital pathways of other suspension feeders [1] because it requires rapid replacement of cells.

The similarity of the feeding cells of sponges, called choanocytes, to unicellular or colonial choanoflagellates (the sister group to animals), coupled with estimates of similar lifespans of 5–6 h [10], suggests replacement occurs by direct replication as in a cell culture. Following such rapid cell proliferation, sponges have been estimated to replace up to one-third of body carbon per day [11], much as in protist cultures [12]. But like other metazoans, sponges have complex tissues whose hallmark is interdependency [13] so populations must be tightly regulated through rate-limiting mechanisms typically relying on stem cells to replenish cell populations (e.g. [14]).

In other animals, cell proliferation is of the order of days rather than hours and is anything but constant. Proliferation varies between different species [15–17] and within an individual depending on food availability [17,18], cell type [19], developmental stage and growth [20,21], and circadian, tidal and seasonal rhythms [22–24]. If cell proliferation rates vary in sponges as they do in other metazoans [25,26], then estimates of carbon transfer through the larger trophic web will also vary.

To determine how universal rapid cell replacement is among sponges, choanocyte proliferation rates were measured in explants of four cold-water sponge species from different taxonomic groups and different life-history characteristics. Factors known to affect cell proliferation in other animals including life-history stage, season, and food availability were examined. Evidence from *in situ* time-lapse imaging suggests that stem cells play a critical role in the routine maintenance of feeding structures in sponges, signifying a major transition from replacement by direct replication. These results illustrate that cell proliferation in sponges is complex, not necessarily rapid and can be accelerated or slowed according to the needs of the organism. They also suggest that the ecological effects caused by the sponge loop come from something other than choanocyte proliferation.

## 2. Material and methods

Four species were selected from different taxonomic groups and with a range of life histories and habitats within British Columbia, Canada (electronic supplementary material, table S1): *Spongilla lacustris* (Class Demospongiae, Order Spongillida), *Sycon coactum* (Class Calcarea, Order Leucosolenida), *Haliclona mollis* (Class Demospongiae, Order Haplosclerida) and *Aphrocallistes vastus* (Class Hexactinellida, Order Hexactinosida). Samples were collected in June and July to estimate choanocyte proliferation rates.

### 2.1. EdU incubations

Initial experiments showed that the sizes of explants and the incubation volumes did not affect 5-ethynyl-2-deoxyuridine (EdU) labelling. Pieces were confirmed to be actively filter feeding by uptake of 1 µm pink fluorescent latex microspheres (Polysciences, CA). Therefore, we used approximately 0.5 cm^3^ explants in 5 ml of water that was replaced daily.

Immediately after collection, pieces approximately 0.5 cm^3^ were cut from the body wall of each of three individuals and edges allowed to heal overnight. Natural variability from individuals collected at the same time was found to be negligible (for *Sp. lacustris* using a test of unequal mean labelling rates; *t *= 0.102, d.f. = 4, *p* = 0.92). Pieces were mixed together and randomly assigned to different incubations in EdU (Life Technologies). EdU incubations were carried out in 5 ml Petri dishes in an incubator to maintain constant temperature and darkness. EdU at a concentration of 100 µM was found to be the lowest concentration producing sufficiently bright and consistent labelling. *Spongilla lacustris* was incubated in 5 ml of 100 µM EdU in 0.2 µm filtered lake water at a typical lake temperature of 18°C. *Sycon coactum*, *H. mollis* and *A. vastus* were incubated in 5 ml of 100 µM EdU in 0.2 µm filtered seawater at 9°C. Sponges were incubated for 6, 8, 12, 16, 18, 20, 24, 30, 36, 48 and 72 h and 4, 5 and 6 days. Length of incubation in EdU varied for each sponge depending on the time at which the maximum number of cells labelled, termed the growth fraction (GF), was reached.

Prior to incubations, water was filtered to avoid differences in proliferation caused by variable food availability; however, to test the effect of feeding activity on choanocyte proliferation, pieces from one individual of *H. mollis* were incubated in EdU in unfiltered (normal) seawater in November 2014 and June 2015. Duplicate water samples were collected from the filtered and unfiltered treatments in June 2015, fixed with 0.15% glutaraldehyde and frozen at −80°C for quantification of bacteria with a FACSCalibur MACPro flow cytometer at the University of Alberta. Cell proliferation of sponges was measured using incubations of EdU as described above.

### 2.2. Sample processing

Sponge explants from all experiments were fixed in 4% paraformaldehyde and 0.03% glutaraldehyde in phosphate-buffered saline (PBS) or Bouin's fixative (*Sy. coactum*) overnight, dehydrated to ethanol, desilicified in 4% hydrofluoric acid and embedded in paraffin wax (Paraplast). Sections 7–10 µm thick were rehydrated in PBS, washed in PBS with 3% bovine serum albumin (wash buffer), permeabilized 2 min in PBS with 0.1% Triton-X (PBTx) and rinsed three times in wash buffer. Sections were incubated in 250 µl of reaction cocktail (Click-iT EdU AlexaFluor 594® Imaging Kit, Life Technologies, Carlsbad, CA, USA) in the dark for 30 min at room temperature, rinsed once with wash buffer and once with PBS. AlexaFluor 488 was used for *Sp. lacustris* to avoid overlap with autofluorescence from symbiotic algae. Nuclei were stained with 100 µM Hoechst 33342 for 30 min; slides were rinsed three times with PBS and mounted with Mowiol.

At least two slides were prepared from each embedded sponge; several sections from each slide were viewed with a Zeiss Axioskop2 Plus microscope and images captured with a QiCam (QImaging) and Northern Eclipse software (Empix Imaging Inc.). Nuclei labelled with EdU (newly synthesized DNA) and nuclei labelled with Hoechst (all nuclei) were counted using the Cell Counter plug-in for Fiji ImageJ [27]. Statistics were calculated using Systat 12 and R.

### 2.3. Calculating cell proliferation

Characteristics of choanocyte proliferation assumed to be proliferating at a steady state were determined by plotting the average proportion of EdU-labelled cells in a choanocyte chamber at each time point, termed the labelling index (LI) [28] (electronic supplementary material, figure S1). The proliferation rate of cells (percent cells labelled h^−1^) indicates the rate at which new cells enter S-phase in their progress through the cell cycle and is the slope of the linear regression of LIs. The *y*-intercept indicates the proportion of cells in S-phase at any given moment (LI_0_). The length of S phase (*T*_s_) is the time interval from the *x*-intercept to the *y*-axis. As incubations increase in length, more and more cells enter S-phase of the cell cycle until all cells that are replicating are labelled, a proportion called the GF. The length of the cell cycle from S-phase to the next round of S-phase (*T*_c_) was calculated as the time until the GF is reached (*T*_c _− *T*_s_) plus the length of time spent in S-phase (*T*_s_). As a result, estimates of cell proliferation rates require at least three time points for a linear regression; cell proliferation values at a single time point can vary greatly depending on the number of cells initially labelled (cells in S-phase) and when no more cells label (the GF) (electronic supplementary material, figure S1*c*).

Statistical comparisons were made between sponges incubated in filtered and unfiltered water. An ANOVA was used to compare the proportions of labelled cells at each time point and an ANCOVA was used to compare the slopes of the proliferation rates calculated.

### 2.4. Microscopy of choanocyte chambers

To understand the mechanism behind cell replacement in a chamber, choanocyte production and replacement was studied in *Sp. lacustris* grown on glass coverslips as described by Elliott & Leys [29]. Choanocyte chambers were located and images captured using a 40× water immersion lens (Zeiss Achroplan on a Zeiss Axioskop2 microscope). Images from each recording were imported as an image stack into ImageJ software and converted to time-lapse video at 25 frames s^−1^ [27,30].

## 3. Results

### 3.1. Cell cycle lengths

**Figure 1.**
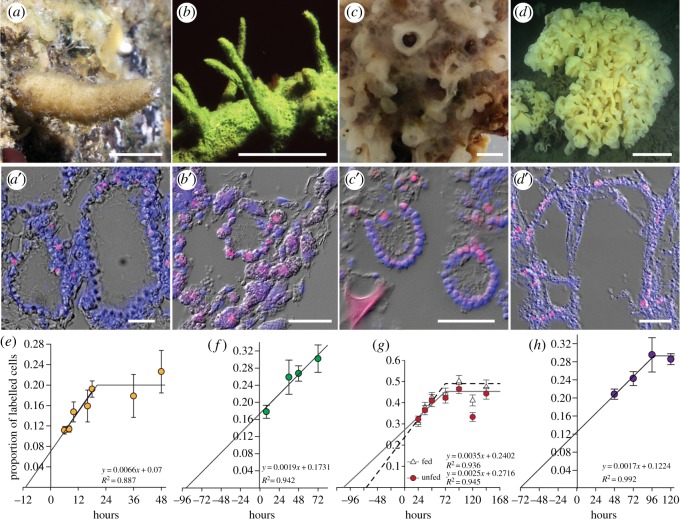
Cell proliferation and cell cycle lengths were measured in EdU-labelled choanocytes from sponge explants with calculations assuming a steady-state choanocyte population. (*a*) *Sycon coactum*; (*b*) *Spongilla lacustris*; (*c*) *Haliclona mollis* sampled in unfiltered water and in filtered water; (*d*) deep-water hexactinellid sponge *Aphrocallistes vastus.* (*a*′–*d*′) New cells (EdU; pink) were counted and compared with total nuclei (Hoechst 33342; blue). Mesohyl cells labelled with EdU are visible in the images of *Sp. lacustris* and *H. mollis*. (*e–h*) Cell cycle lengths determined assuming a steady-state population of cells, after Nowakowski *et al*. [28]. (*e*) *Sy. coactum*; (*f*) *Sp. lacustris*; (*g*) *H. mollis* incubated in unfiltered (fed) and filtered (unfed) water; (*h*) *A. vastus*. Scale bars: (*a*–*c*) 1 cm; (*d*) 10 cm; (*a*′–*d*′) 25 µm.

Cell cycle lengths in explants of the four species studied ranged from 30 to 170 h, longer than any measured previously [10,31]. The cell cycle was shortest in the calcareous sponge *Sy. coactum* at 30 h (table 1 and figure 1) and much longer (more than 4 days) in adult *Sp. lacustris, H. mollis* and *A. vastus* (table 1 and figure 1).

**Table 1.** Characteristics of choanocyte populations from explants of four species of cold-water sponges. Proportions of cells in S phase at a given time, the rate of proliferation, asymptotic/maximum proportion of proliferating cells in a population (growth fraction, GF), estimated length of S phase (*T*_s_) and estimated length of cell cycle (*T*_c_).

	*Sycon coactum*	*Spongilla lacustris* (gemmules)^a^	*Spongilla lacustris* (adult)	*Haliclona mollis*^b^	*Aphrocallistes vastus*
cells in S phase (%)	7.1 ± 1.7	6.6 ± 6.8	17 ± 1.6	27 ± 2.1	12 ± 1.2
proliferation rate (% cells h^−1^)	0.7 ± 0.1	2.0 ± 0.36	0.19 ± 0.03	0.25 ± 0.04	0.17 ± 0.02
growth fraction (GF; %)	20 ± 1.4	70 ± 9.8	30 ± 1.5	44 ± 1.5	29 ± 0.5
length of S phase (*T*_s_; h)	10.9	3.2	91	108	72
cell cycle length (*T*_c_; h)	30.7	34.6	≥159	176	170

^a^For full-size, central chambers in gemmules incubated 5 days post-hatching. Smaller peripheral chambers had 16.8 ± 4.6% of cells in S phase, proliferation rate of 2.0 ± 0.3% cells h^−1^, 73 ± 5.2% of cells as the GF, 8.3 h *T*_s_ and 36.2 h *T*_c_.

^b^For *H. mollis* in filtered seawater. *H. mollis* incubated in unfiltered seawater had 24.0 ± 3.1% of cells in S phase, proliferation rate of 0.25 ± 0.04% cells h^−1^, 48 ± 2.3% of cells as the GF, 69.2 h *T*_s_ and 138 h *T*_c_.

Cell types in the tissues were not equally proliferative. Choanocytes and amoeboid cells from the mesohyl labelled with equal frequency in *H. mollis*, whereas two to five times more choanocytes were labelled than mesohyl cells in *Sp. lacustris*. Outer epithelial cells (pinacocytes) labelled after 72 h in *Sp. lacustris* and *H. mollis* but never labelled in *Sy. coactum* or *A. vastus* even after 4 days. For *Sp. lacustris* more choanocytes labelled in small chambers near the periphery than in full-sized chambers near the centre of the sponge yet cell cycle lengths were nearly identical (table 1; electronic supplementary material, figure S2).

### 3.2. Factors affecting cell proliferation

Rates of cell replacement varied with season, life-history stage and body region. Sponges collected in summer months had three times as many proliferating cells than those collected later in the year (*Sy. coactum* 18 ± 4% in June compared with 4.5 ± 1.4% in August) and during winter months hardly any cells were replaced (*H. mollis*). Cell proliferation rates were 10 times faster in newly hatched sponges than in adult tissues of *Sp. lacustris* measured from explants (figure 1; electronic supplementary material, figure S2). The cell cycle length in growing regions of a deep-sea glass sponge *A. vastus* was 170 h, but nuclei seldom labelled in mature regions of the tissue and then only after 4 days (table 1 and figure 1).

To determine whether food availability and food handling could alter cell replacement rates, we exposed *H. mollis* to different concentrations of food. Proliferation rates were not significantly different between the two treatments (table 1, figure 1) (proliferation rate: ANCOVA, *F* = 1.0367, *p* = 0.309; proportions of labelled cells: ANOVA, *F* = 0.687, *p* = 0.407). However, explants in water with more bacteria had a shorter estimated cell cycle length (138 h in normal seawater compared with 176 h in filtered seawater) (table 1 and figure 1) which may suggest that feeding activity affects cell proliferation in the long term. In contrast however, choanocytes in sterile cultured *Sp. lacustris* showed a much higher proliferation rate than sponges grown in non-sterile lake water (table 1, figure 1). These contrasting observations suggest that cell turnover is more complex than simple exhaustion and replacement of cells due to feeding activity.

### 3.3. Video microscopy of choanocyte chambers

**Figure 2.**
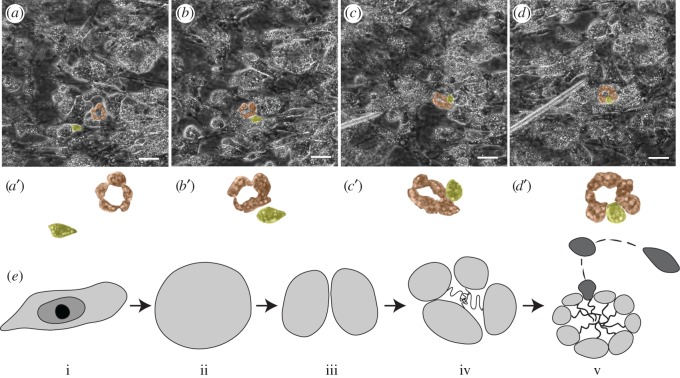
Immigration and differentiation of mesohyl cells into mature choanocyte chambers. *In situ* time-lapse microscopy of *Spongilla lacustris*. (*a*,*a*′) An amoeboid cell (yellow) moves through the mesohyl, (*b*,*b*′) it slows and changes direction towards two small choanocyte chambers (orange). (*c*,*c*′) It moves against one of the choanocyte chambers, stops and inserts itself between the choanocytes, (*d*, *d*′) changes shape to resemble that of other choanocytes in the chamber. (*e*) Schematic of the different steps of chamber formation and maintenance. An archaeocyte (i) undergoes several mitotic divisions (ii, iii) to make up the foundation of the chamber (iv), while choanocytes are replaced by differentiation of stem cells from the mesohyl that immigrate into the chamber (v) (dark grey). Scale bars: 25 µm.

To determine how choanocytes proliferate and are replaced in a chamber, we filmed small and large choanocyte chambers *in situ* in the thin tissue of recently hatched freshwater sponges. Remarkably, the flagellated chambers used for feeding moved freely through the mesohyl as a unit until plumbed into a canal and whole chambers were seen being tugged by a mesohyl cell (electronic supplementary material, figure S3 and video S1). Importantly, while new chambers formed by division of a single cell to form two to six cells, in larger chambers choanocytes did not divide during the time of observation. Instead, amoeboid cells moving through the mesohyl stopped and turned towards a choanocyte chamber they had passed, eventually squeezing between the other cells to become a choanocyte (figure 2; electronic supplementary material, video S2). These ‘immigrant’ choanocytes formed elsewhere from a stem cell population represent a previously unknown yet seemingly common method of choanocyte formation and replacement in mature chambers of cellular sponges.

## 4. Discussion

Ocean habitats are affected by global change, from fishing to eutrophication to climate change. Understanding the mechanism by which suspension feeders affect surrounding communities through carbon transfer is therefore important. The sponge loop represents a new and potentially major component of carbon flow but requires a thorough understanding of different sponges' cell biology and ecology. The variability in cell proliferation both between species and within an individual that we describe here for cold-water species suggests that the sponge loop functions through mechanisms shared with other suspension feeding animals (e.g. excretion of wastes [32,33]) rather than through cell shedding. Sponges can have a range of cell cycle lengths depending on life-history stages, growth and season suggesting food availability is important. Importantly, cell replacement in sponge feeding epithelia occurs via stem cells, not direct replication, which suggests complex regulation of tissue homeostasis as in other metazoans.

### 4.1. Variability of cell replacement rates

**Figure 3.**
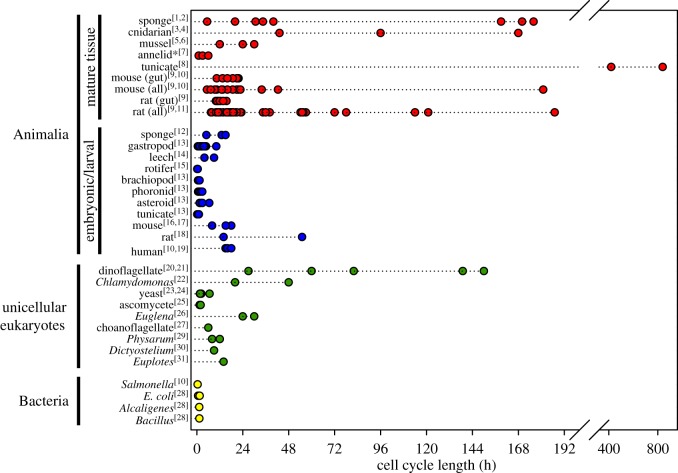
Cell cycle lengths (*T*_c_) for cell types in unicells and metazoans. Points show the range reported in hours (data and references provided in electronic supplementary material, table S2). Mature animal tissues have a wide range of cell cycle lengths but they are typically several days longer than those in unicellular eukaryotes and prokaryotes. A notable exception is embryonic and larval tissues of metazoans, which have much shorter cell cycle lengths.

Our results show that cell replacement rates in sponges can vary between species and even change across one species' lifespan, and with season. Cell cycle lengths are similar to those of other metazoans, ranging from 5 h to over 7 days [10,31] (figure 3; electronic supplementary material, table S2). By contrast, the unicellular flagellate *Monosiga brevicollis* replaces cells every 6 h during log phase of growth in a fed culture [34], and other unicellular cultures are generally shorter (figure 3; electronic supplementary material, table S2). In a multicellular animal, different cell types have proliferation rates depending on their functions. The fastest rates measured from animal tissues were 1–6 h in the trophosomes of worms and bivalves from hydrothermal vents and cold seeps [35], gut epithelia in mice were replaced every 10–22 h [36], and the slowest rates were up to several weeks in the endostyle and oesophagus of the ascidian *Styela clava* [37].

Proliferative activity was seasonal and varied with life history. In *H. mollis*, where in summer the proliferation rate was 0.25 cells h^−1^, in winter months too few cells labelled to calculate any cell kinetics. Sponges with short life histories (1 year for *Sy. coactum*, *Sp. lacustris* and *Hymeniacidon sinapium* [31,38,39]) have comparable cell cycle lengths of 20–40 h, compared with several days for species living many years. The relatively rapid cell cycle length of choanocytes in these species with a short life history may contribute to their success as seasonal or invasive species [38] and may optimize the active season for freshwater sponges [40]. Temperature may also have an effect, as *Halisarca caerulea* [10] is a tropical sponge and has the fastest cell replacement rate (5.4 h) of any sponge studied so far.

Other considerations that may affect calculations of cell cycle length include methodology such as the type of tracer used (5-bromo-2-deoxyuridine, which can be teratogenic, mutagenic and a trigger of cell death (reviewed by [41]), EdU or ^3^H-thymidine), feeding or wounding [25]. Unlike mouse gut epithelia, sponge choanocytes generate a feeding current by beating a flagellum and phagocytose particulate food, but food handling does not appear to be the major cost because cell cycle length was not significantly shorter in sponges grown in high concentrations of bacteria. Sponges grown in water supplemented with 2 × 10^6^ cells ml^−1^ heat-killed bacteria produced a cloud of waste material around the osculum, but showed no change in cell proliferation (electronic supplementary material, figure S4). Alexander *et al*. [25] showed that cell replacement rates varied with distance from a wound edge in *Halisarca caerulea*. How broadly applicable this is remains unknown, but it was suggested that energy is redirected from cell replacement to wound healing processes. Repair from damage is typically thought to be rapid (overnight recovery from wounding [42]) though it can take several weeks [43]. Although we did not see morphological evidence of wound healing, if the energy budget was displaced to heal the explants we used, this might explain the lower rate of cell replacement in our study compared with those using fully intact sponges. However, we found too few cells labelled in 6 h to determine cell replacement kinetics, even on a freshwater sponge cultured at room temperature. Therefore, we used small pieces (explants) with exchanges of sufficient volume to allow filtration by the pieces over multiple time points.

Comparing cell replacement among species assumes that all cells labelled are in a steady state. Here it assumes that cell numbers in sponge flagellated chambers reach a terminal size [44], but as shown above this assumption could easily be violated during growth or repair [45]. Growing chambers occur when a sponge is first forming and also along edges during its life and during rearrangements of the choanosome that occur seasonally and during reproduction [45–47]. The ‘one population’ steady-state method of Nowakowski *et al*. [28] relies on knowing two characteristics, the maximum number of cells that label and the time in which that happens. The proportion of labelled cells measured at a single time point (e.g. [48]) does not indicate cell proliferation rates and could reflect different cell cycle lengths and growth fractions (electronic supplementary material, figure S1*c*). Equally, if cells are not in steady state due to wounding or growth or seasonal feeding patterns, then the proportion of cells that label (GF) over time will change.

### 4.2. Sources of choanocytes in mature choanocyte chambers

Surprisingly little is known about how choanocytes form. For newly forming chambers choanocytes are thought to arise from several divisions of a single archaeocyte [44,49] or from transdifferentiation of larval ciliated epithelial cells into choanocytes, possibly with an intermediate step as archaeocytes [50,51]. Clearly, the first choanocytes form by mitosis, but there is no evidence that this happens in mature chambers given the small size of cells and limited ability to divide because of machinery tied into the flagellum [52], low nucleic acid content and no visible evidence of mitosis despite plenty of cells labelled by EdU. Choanocyte chambers move as a single unit and as they are maintained by a stem cell population of cells they could be considered to be a sponge tissue. Although it had been shown previously that they can be inserted, ready-made, into canals [53], ours is the first work to show they form and are maintained by a stem cell population of mesohyl cells. Pinacocytes also do not divide and must originate from cells in the mesohyl that migrate into the epithelium [45,49]. Rates of replacement of choanocyte and pinacocyte populations are therefore at least partially driven by the rate that stem cells from the mesohyl immigrate rather than by direct proliferation, and this is further evidence that the steady-state model of Nowakowski *et al*. [28] does not properly apply to these kinds of epithelia. And so for sponges, it may be most appropriate to compare proliferation rate (the slope) rather than cell cycle length or growth fraction (maximum number of cells that label) because proliferation rates are independent of any assumptions of a steady-state population [28].

### 4.3. Ecological implications of variable cell turnover

**Figure 4.**
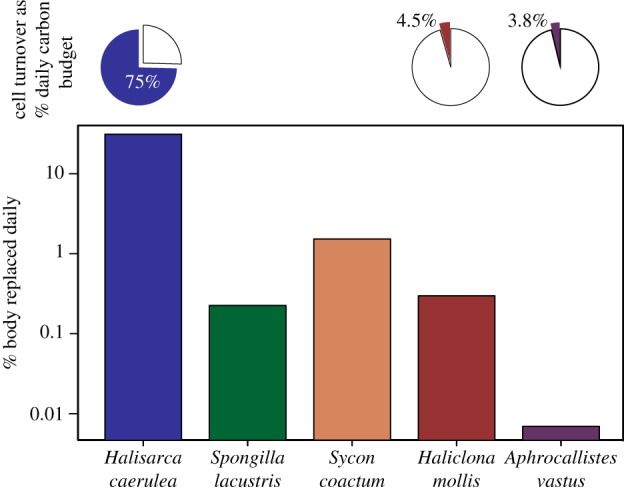
Cell replacement as a proportion of the carbon budget and body volume of sponges. The percent of the body volume that is replaced by sponges each day (bars), and the proportion of carbon consumed that is used in replacing cells (pie charts) (electronic supplementary material, tables S3 and S4) for *Halisarca caerulea* and for explants from *H. mollis* and *A. vastus*.

The cells of a sponge function as a cooperative unit to grow, feed, reproduce and senesce. Changes in cell proliferation rates reflect a physiological plasticity to changing conditions that will vary the mechanism by which sponges transfer carbon. If cells are shed at the same rate they are produced, then a sponge like *Halisarca caerulea* would lose (and replace) 31% of its body volume each day (electronic supplementary material, table S3). In comparison, the cold-water sponge explants we studied replaced 0.01–1.5% of their body volume each day (electronic supplementary material, table S3) [10]. Using estimates of carbon lost due to choanocyte shedding [10] and carbon uptake [54] (summarized in [55]), this amount of turnover in *Halisarca* comes at an energetic cost of 75% of the daily food energy (DOC and POC) consumed, compared with less than 5% of the total food energy consumed for *H. mollis* and *A. vastus* (figure 4; electronic supplementary material, tables S3 and S4). The range of these processes in different sponges suggests different mechanisms of carbon transfer in different regions. In temperate (cold-water) regions, it is likely that excretion of waste drives the connection sponges make between microbes in the water column and benthic biomass [32].

## Supplementary Material

Supplemental materials.pdf: Supplemental materials including Supplemental Figures 1-4, Supplemental Tables 1-4, captions for Supplemental Videos 1-2, and Supplemental methods.

## Data Availability

Data are deposited at Dryad: http://dx.doi.org/10.5061/dryad.1787f [56].

## References

[1] Gili J-M, Coma R 1998 Benthic suspension feeders: their paramount role in littoral marine food webs. Trends Ecol. Evol. 13, 316–321. (doi:10.1016/S0169-5347(98)01365-2) 21238320 10.1016/s0169-5347(98)01365-2

[2] De Goeij JM, van Oevelen D, Vermeij MJA, Osinga R, Middelburg JJ, de Goeij AFPM, Admiraal W 2013 Surviving in a marine desert: the sponge loop retains resources within coral reefs. Science 342, 108–110. (doi:10.1126/science.1241981) 24092742 10.1126/science.1241981

[3] Pawlik JR, Burkepile DE, Thurber RV 2016 A vicious circle? Altered carbon and nutrient cycling may explain the low resilience of Caribbean coral reefs. Bioscience 66, 470–476. (doi:10.1093/biosci/biw047)

[4] Silveira CB et al. 2015 Microbial and sponge loops modify fish production in phase-shifting coral reefs. Environ. Microbiol. 17, 3832–3846. (doi:10.1111/1462-2920.12851) 25817914 10.1111/1462-2920.12851

[5] Leal MC, Sheridan C, Osinga R, Dionísio G, Rocha RJ, Silva B, Rosa R, Calado R 2014 Marine microorganism-invertebrate assemblages: perspectives to solve the ‘supply problem’ in the initial steps of drug discovery. Mar. Drugs 12, 3929–3952. (doi:10.3390/md12073929) 24983638 10.3390/md12073929 PMC4113807

[6] Rivera AS et al. 2011 RNA interference in marine and freshwater sponges: actin knockdown in *Tethya wilhelma* and *Ephydatia muelleri* by ingested dsRNA expressing bacteria. BMC Biotechnol. 11, 67 (doi:10.1186/1472-6750-11-67) 21679422 10.1186/1472-6750-11-67 PMC3146823

[7] Woodland HR 2016 The birth of animal development: multicellularity and the germline. In Current topics in developmental biology (ed. Paul MW), pp. 609–630. New York, NY: Academic Press. 10.1016/bs.ctdb.2015.10.020 26970004

[8] Wijgerde T 2015 Experimental aquaculture of Dendronephthya corals. Adv. Aquarist 16 (http://www.advancedaquarist.com/2015/7/aafeature)

[9] Aktipis CA, Boddy AM, Jansen G, Hibner U, Hochberg ME, Maley CC, Wilkinson GS 2015 Cancer across the tree of life: cooperation and cheating in multicellularity. Phil. Trans. R. Soc. B 370, 20140219 (doi:10.1098/rstb.2014.0219) 26056363 10.1098/rstb.2014.0219 PMC4581024

[10] De Goeij JM, De Kluijver A, Van Duyl FC, Vacelet J, Wijffels RH, De Goeij AFPM, Cleutjens JPM, Schutte B 2009 Cell kinetics of the marine sponge *Halisarca caerulea* reveal rapid cell turnover and shedding. J. Exp. Biol. 212, 3892–3900. (doi:10.1242/jeb.034561) 19915132 10.1242/jeb.034561

[11] Rix L et al. 2016 Coral mucus fuels the sponge loop in warm- and cold-water coral reef ecosystems. Sci. Rep. 6, 18715 (doi:10.1038/srep18715) 26740019 10.1038/srep18715 PMC4703987

[12] Fenchel T 1982 Ecology of heterotrophic microflagellates. II. Bioenergetics and growth. Mar. Ecol. Prog. Ser. 8, 225–231. (doi:10.3354/meps008225)

[13] Grosberg RK, Strathmann RR 2007 The evolution of multicellularity: a minor major transition? Annu. Rev. Ecol. Evol. Syst. 38, 621–654. (doi:10.1146/annurev.ecolsys.36.102403.114735)

[14] Newmark PA, Sánchez Alvarado A 2000 Bromodeoxyuridine specifically labels the regenerative stem cells of planarians. Dev. Biol. 220, 142–153. (doi:10.1006/dbio.2000.9645) 10753506 10.1006/dbio.2000.9645

[15] David CN 1983 Cell cycle analysis of *Hydra* cells. In Hydra: research methods (ed. Lenhoff HM), pp. 157–164. New York, NY: Plenum Press.

[16] Holstein TW, David CN 1990 Cell cycle length, cell size, and proliferation rate in *Hydra* stem cells. Dev. Biol. 142, 392–400. (doi:10.1016/0012-1606(90)90360-U) 2257972 10.1016/0012-1606(90)90360-u

[17] Otto JJ, Campbell RD 1977 Tissue economics of *Hydra*: regulation of cell cycle, animal size and development by controlled feeding rates. J. Cell Sci. 28, 117–132. 599169 10.1242/jcs.28.1.117

[18] Bosch TC, David CN 1984 Growth regulation in *Hydra*: relationship between epithelial cell cycle length and growth rate. Dev. Biol. 104, 161–171. (doi:10.1016/0012-1606(84)90045-9) 6734933 10.1016/0012-1606(84)90045-9

[19] Pellettieri J, Alvarado AS 2007 Cell turnover and adult tissue homeostasis: from humans to planarians. Annu. Rev. Genet. 41, 83–105. (doi:10.1146/annurev.genet.41.110306.130244) 18076325 10.1146/annurev.genet.41.110306.130244

[20] Cheng H, Leblond CP 1974 Origin, differentiation and renewal of the four main epithelial cell types in the mouse small intestine I. Columnar cell. Am. J. Anat. 141, 461–479. (doi:10.1002/aja.1001410403) 4440632 10.1002/aja.1001410403

[21] Alexiades MR, Cepko C 1996 Quantitative analysis of proliferation and cell cycle length during development of the rat retina. Dev. Dyn. 205, 293–307. (doi:10.1002/(SICI)1097-0177(199603)205:3<293::AID-AJA9>3.0.CO;2-D) 8850565 10.1002/(SICI)1097-0177(199603)205:3<293::AID-AJA9>3.0.CO;2-D

[22] Zaldibar B, Cancio I, Marigómez I 2004 Circatidal variation in epithelial cell proliferation in the mussel digestive gland and stomach. Cell Tissue Res. 318, 395–402. (doi:10.1007/s00441-004-0960-0) 15503161 10.1007/s00441-004-0960-0

[23] Chisholm SW, Vaulot D, Olson RJ 1984 Cell cycle controls in phytoplankton. In Cell cycle clocks (ed. Edmunds LN Jr), pp. 365–394. New York, NY: Marcel Dekker, Inc.

[24] Leibson NL, Frolova LT 1994 Winter-spring essential reorganization of cell proliferation in the digestive tract epithelia in the mussel *Crenomytilus grayanus*. Mar. Biol. 118, 471–477. (doi:10.1007/BF00350304)

[25] Alexander BE, Achlatis M, Osinga R, van der Geest HG, Cleutjens JPM, Schutte B, de Goeij JM 2015 Cell kinetics during regeneration in the sponge *Halisarca caerulea*: how local is the response to tissue damage? PeerJ 3, e820 (doi:10.7717/peerj.820) 25780772 10.7717/peerj.820 PMC4358696

[26] Alexander BE, Mueller B, Vermeij MJA, van der Geest HHG, de Goeij JM 2015 Biofouling of inlet pipes affects water quality in running seawater aquaria and compromises sponge cell proliferation. PeerJ 3, e1430 (doi:10.7717/peerj.1430) 26664799 10.7717/peerj.1430 PMC4675111

[27] Schindelin J et al. 2012 Fiji: an open-source platform for biological-image analysis. Nat. Methods 9, 676–682. (doi:10.1038/nmeth.2019) 22743772 10.1038/nmeth.2019 PMC3855844

[28] Nowakowski RS, Lewin SB, Miller MW 1989 Bromodeoxyuridine immunohistochemical determination of the lengths of the cell cycle and the DNA-synthetic phase for an anatomically defined population. J. Neurocytol. 18, 311–318. (doi:10.1007/BF01190834) 2746304 10.1007/BF01190834

[29] Elliott GRD, Leys SP 2007 Coordinated contractions effectively expel water from the aquiferous system of a freshwater sponge. J. Exp. Biol. 210, 3736–3748. (doi:10.1242/jeb.003392) 17951414 10.1242/jeb.003392

[30] Schneider CA, Rasband WS, Eliceiri KW 2012 NIH image to ImageJ: 25 years of image analysis. Nat. Methods 9, 671–675. (doi:10.1038/nmeth.2089) 22930834 10.1038/nmeth.2089 PMC5554542

[31] Shore RE 1971 Growth and renewal studies of the choanocyte population in *Hymeniacidon sinapium* (Porifera: Demospongiae) using Colcemid and 3-H thymidine. J. Exp. Biol. 177, 359–363. (doi:10.1002/jez.1401770310)

[32] Wolfrath B, Barthel D 1989 Production of faecal pellets by the marine sponge *Halichondria panicea* Pallas (1766). J. Exp. Mar. Biol. Ecol. 129, 81–94. (doi:10.1016/0022-0981(89)90064-6)

[33] Kahn AS 2016 Ecophysiology of glass sponge reefs. PhD thesis, University of Alberta, Edmonton, Alberta, Canada.

[34] King N, Hittinger CT, Carroll SB 2003 Evolution of key cell signaling and adhesion protein families predates animal origins. Science 301, 361–363. (doi:10.1126/science.1083853) 12869759 10.1126/science.1083853

[35] Pflugfelder B, Cary SC, Bright M 2009 Dynamics of cell proliferation and apoptosis reflect different life strategies in hydrothermal vent and cold seep vestimentiferan tubeworms. Cell Tissue Res. 337, 149–165. (doi:10.1007/s00441-009-0811-0) 19444472 10.1007/s00441-009-0811-0

[36] Van't Hof J 1965 Relationships between mitotic cycle duration, S period duration and the average rate of DNA synthesis in the root meristem cells of several plants. Exp. Cell Res. 39, 48–58. (doi:10.1016/0014-4827(65)90006-6) 5831250 10.1016/0014-4827(65)90006-6

[37] Ermak TH 1975 Cell proliferation in the digestive tract of *Styela clava* (Urochordata: Ascidiacea) as revealed by autoradiography with tritiated thymidine. J. Exp. Zool. 194, 449–465. (doi:10.1002/jez.1401940302) 10.1016/0040-8166(76)90007-0 988647

[38] Cao H, Cao X, Guan X, Xue S, Zhang W 2012 High temporal variability in bacterial community, silicatein and hsp70 expression during the annual life cycle of *Hymeniacidon sinapium* (Demospongiae) in China's Yellow Sea. Aquaculture 358–359, 262–273. (doi:10.1016/j.aquaculture.2012.06.005)

[39] Eerkes-Medrano DI, Leys SP 2006 Ultrastructure and embryonic development of a syconoid calcareous sponge. Invert. Biol. 125, 177–194. (doi:10.1111/j.1744-7410.2006.00051.x)

[40] Frost TM 1982 Population dynamics and standing biomass of the freshwater sponge *Spongilla lacustris*. Ecology 63, 1203–1210. (doi:10.2307/1938844)

[41] Taupin P 2007 BrdU immunohistochemistry for studying adult neurogenesis: paradigms, pitfalls, limitations, and validation. Brain Res. Rev. 53, 198–214. (doi:10.1016/j.brainresrev.2006.08.002) 17020783 10.1016/j.brainresrev.2006.08.002

[42] Ayling AL 1983 Growth and regeneration rates in thinly encrusting Demospongiae from temperate waters. Biol. Bull. 165, 343–352. (doi:10.2307/1541200) 10.2307/1541200 28368226

[43] Duckworth AR 2003 Effect of wound size on the growth and regeneration of two temperate subtidal sponges. J. Exp. Mar. Biol. Ecol. 287, 139–153. (doi:10.1016/S0022-0981(02)00552-X)

[44] Tanaka K, Watanabe Y 1984 Choanocyte differentiation and morphogenesis of choanocyte chambers in the fresh-water sponge, *Ephydatia fluviatilis*, after reversal of developmental arrest caused by hydroxyurea. Zool. Sci. 1, 561–570.

[45] Simpson TL 1984 The cell biology of sponges. New York, NY: Springer.

[46] Harrison FW 1974 Histology and histochemistry of developing outgrowths of *Corvomeyenia carolinensis* Harrison (Porifera: Spongillidae). J. Morphol. 144, 185–194. (doi:10.1002/jmor.1051440205) 10.1002/jmor.1051440205 30322226

[47] Gilis M, Gosselin P, Dubois P, Willenz P 2011 Seasonal modifications and morphogenesis of the hypercalcified sponge *Petrobiona massiliana* (Calcarea, Calcaronea). Invert. Biol. 130, 193–210. (doi:10.1111/j.1744-7410.2011.00239.x)

[48] Alexander BE et al. 2014 Cell turnover and detritus production in marine sponges from tropical and temperate benthic ecosystems. PLoS ONE 9, e109486*.* (doi:10.1371/journal.pone.0109486) 25289641 10.1371/journal.pone.0109486 PMC4188633

[49] Brien P 1976 La croissance des Spongillidae. Formation des choanocytes et des spicules. Bull. Biol. France Belg. 110, 211–252.

[50] Amano S, Hori I 1996 Transdifferentiation of larval flagellated cells to choanocytes in the metamorphosis of the demosponge *Haliclona permollis*. Biol. Bull. 190, 161–172. (doi:10.2307/1542536) 10.2307/1542536 29244590

[51] Leys SP, Degnan BM 2002 Embryogenesis and metamorphosis in a haplosclerid demosponge: gastrulation and transdifferentiation of larval ciliated cells to choanocytes. Invert. Biol. 121, 171–189. (doi:10.1111/j.1744-7410.2002.tb00058.x)

[52] King N 2004 The unicellular ancestry of animal development. Dev. Cell 7, 313–325. (doi:10.1016/j.devcel.2004.08.010) 15363407 10.1016/j.devcel.2004.08.010

[53] Weissenfels N 1981 Bau und Funktion des Süßwasserschwamms *Ephydatia fluviatilis* L. (Porifera). Zoomorphology 98, 35–45. (doi:10.1007/BF00310319)

[54] De Goeij JM, Van Den Berg H, van Oostveen MM, Epping EEHG, van Duyl FCF 2008 Major bulk dissolved organic carbon (DOC) removal by encrusting coral reef cavity sponges. Mar. Ecol. Prog. Ser. 357, 139–151. (doi:10.3354/meps07403)

[55] Maldonado M, Ribes M, Van Duyl FC 2012 Nutrient fluxes through sponges: biology, budgets, and ecological implications. Adv. Mar. Biol. 62, 113–182. (doi:10.1016/B978-0-12-394283-8.00003-5) 22664122 10.1016/B978-0-12-394283-8.00003-5

[56] Kahn AS, Leys SP 2016 Data from: The role of cell replacement in benthic--pelagic coupling by suspension feeders. Dryad Digital Repository. (doi:10.5061/dryad.1787f) 10.1098/rsos.160484 PMC5180130 28018632

