# Supplementary material for: The role of cell replacement in benthic–pelagic coupling by suspension feeders
Source: R Soc Open Sci. 2016 Nov 30;3(11):160484. doi: 10.1098/rsos.160484 (PMC5180130; doi:10.1098/rsos.160484)
Supplement: Supplemental materials.pdf: Supplemental materials including Supplemental Figures 1-4, Supplemental Tables 1-4, captions for Supplemental Videos 1-2, and Supplemental methods. [file rsos160484supp1.pdf]

Supplemental materials

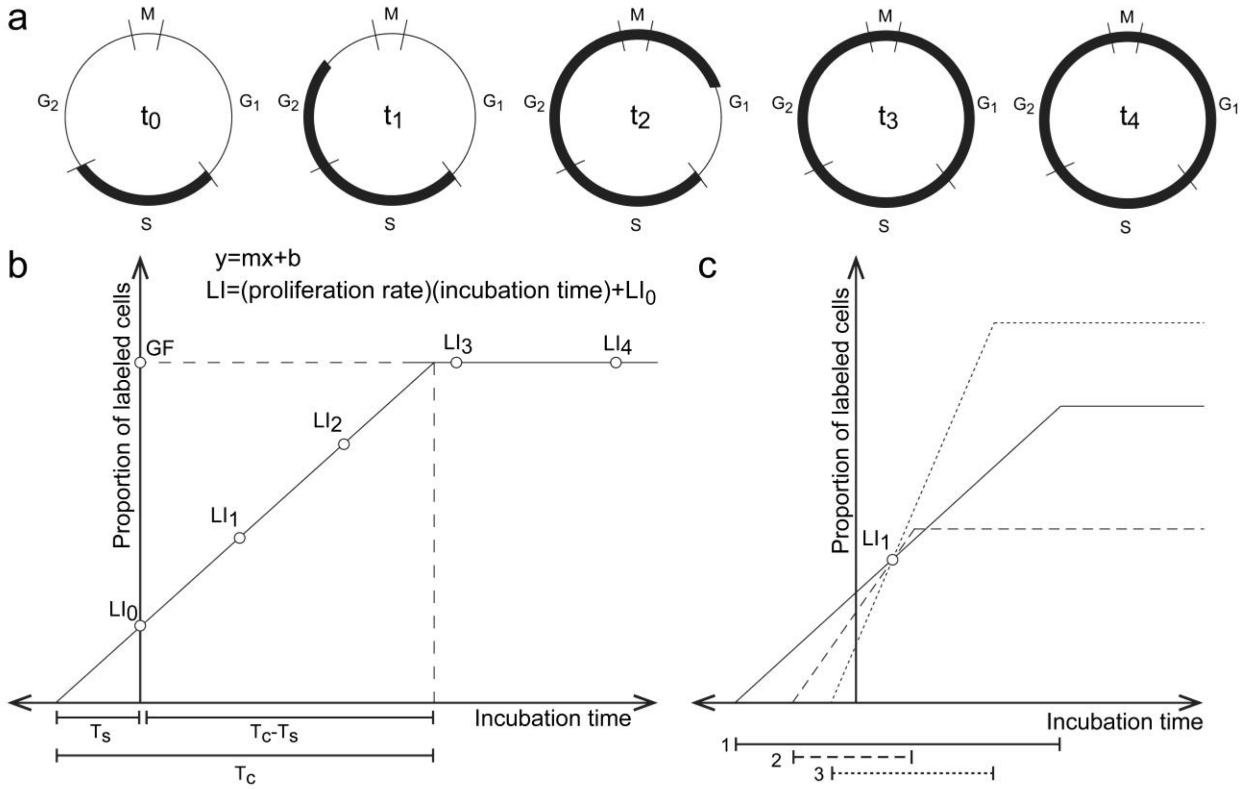

Supplemental Figure 1. Theoretical model for labeling steady state cell populations, after Nowakowski 1989. A: The proportion of EdU-labeled nuclei increases with time as more cells enter S-phase of the cell cycle, until all cells in a population have passed through S-phase. B: EdU incubations of a steady state population for different lengths of time result in labeling indices (LI) along a linear curve that eventually levels off when the growing fraction of cells (GF) has all passed through a complete cell cycle. The y-intercept corresponds with the proportion of cells in S-phase at a given moment and is back-calculated from the regression line. The time from the x-intercept to the ordinate axis is the calculated length of S phase (T_s). The time from the x-intercept to the asymptote is the length of the cell cycle (T_c). **C:** A single time point (LI) cannot be used to compare cell cycle kinetics between species or treatments because of the different cell cycle lengths and GF that result from different slopes (proliferation rates).

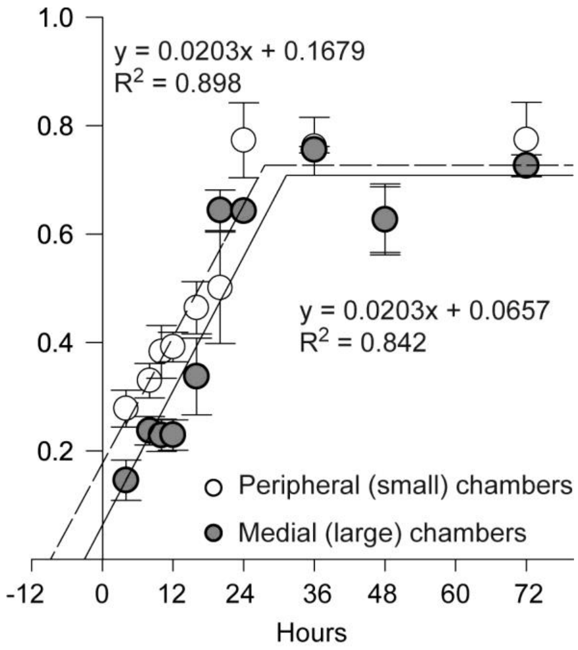

Supplemental Figure 2. Proportions of EdU-labeled choanocytes with calculations assuming a steady state choanocyte population in fully grown, central choanocyte chambers and in smaller, still-forming chambers at the periphery of *Spongilla lacustris* hatched from gemmules. Error bars indicate standard error.

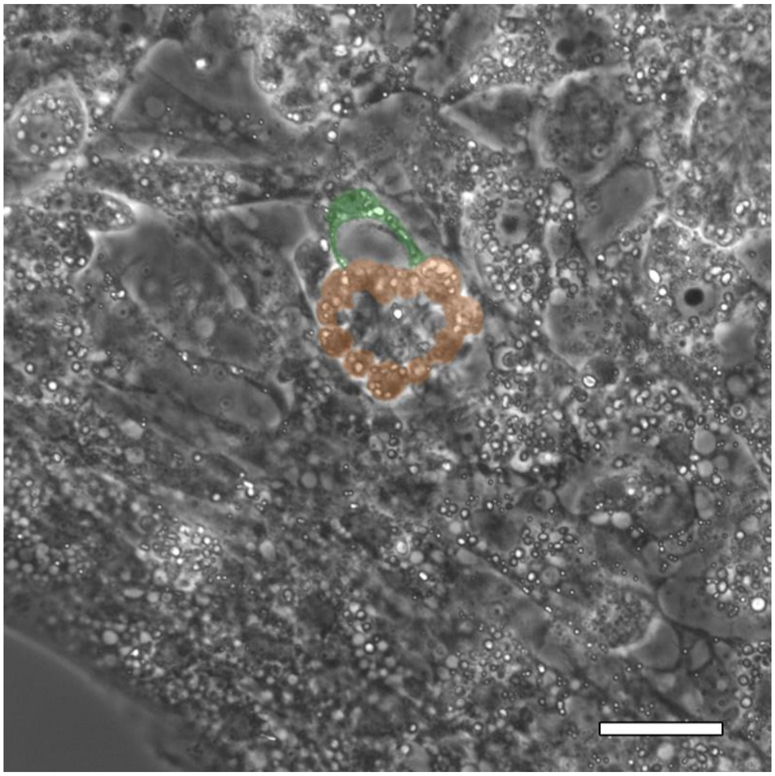

Supplemental Figure 3. Choanocytes move as a unit and in this instance a choanocyte chamber (pseudocoloured orange) was being pulled by a cell from the mesohyl that attached to it with two pseudopodia (pseudocoulored green). A time-lapse video of this is shown in Supplemental Video 1. Scale bar: 25 µm.

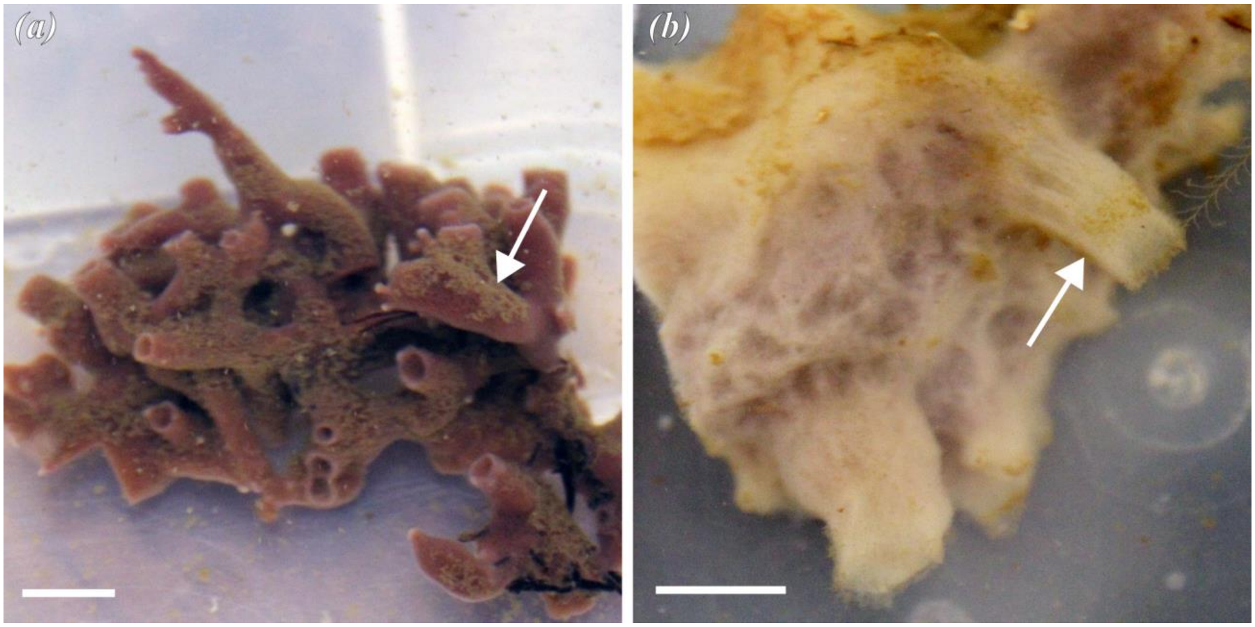

Supplemental Figure 4. Sponges incubated in bacteria-enriched seawater produced brown detritus in their tanks whereas those in filtered seawater produced less detritus. A. *Haliclona permollis*, an intertidal demosponge. B. *Haliclona mollis*, a subtidal demosponge. Arrows point to the brown detritus, which was often found near the oscula of the sponges. Scale bars: 1 cm.

Supplemental Table 1. Life histories of four sponge species from different habitats.

Species	Class	Habitat	Life history
Aphrocallistes vastus	Hexactinellida	>100 m	Multi-year
Haliclona mollis	Demospongiae	Subtidal	Multi-year
Spongilla lacustris[*]	Demospongiae	Freshwater	Annual
Sycon coactum	Calcarea	Subtidal	Annual

[*]Both mature sponges and overwintering cysts (gemmules) were collected and their cell proliferation compared.

Supplemental Table 2. Cell cycle lengths harvested from the literature for a variety of cell types from mature and larval/embryonic animals, unicellular eukaryotes, bacteria, and syncytial tissues. Relevant notes are listed in the notes section. References match those listed in Figure 3.

Species	Kingdom	group	cell type	T_c (h)	Notes	Reference
**Mature animal tissue**						
Spongilla lacustris	Animalia	Porifera	choanocyte	≥159		this study
Sycon coactum	Animalia	Porifera	choanocyte	30.2		this study
Haliclona mollis	Animalia	Porifera	choanocyte	176		this study
Aphrocallistes vastus	Animalia	Porifera	choanoblast	170		this study
Spongilla lacustris (gemmule)	Animalia	Porifera	choanocyte	34.3		this study
Hymeniacidon sinapium	Animalia	Porifera	choanocyte	20-40		[1]
Halisarca caerulea	Animalia	Porifera	choanocyte	5.4		[2]
Hydra attenuata	Animalia	Cnidaria	epithelial cell	96-168	Longer cell cycle during food-poor or starved conditions.	[3]
Hydra oligactis	Animalia	Cnidaria	interstitial stem cells	43.2-96		[4]
Mytilus galloprovincialis	Animalia	Mollusca, Bivalvia	gill cell	24-30		[5]
Mytlius galloprovincialis	Animalia	Mollusca, Bivalvia	epithelia (stomach & digestive gland)	12	Tides drive rhythm of cell cycle.	[6]
Riftia pachyptila	Animalia	Annelida	epidermis	6	Hydrothermal vent worm	[7]
Riftia pachyptila	Animalia	Annelida	peripheral trophosome	6	Hydrothermal vent worm	[7]
Riftia pachyptila	Animalia	Annelida	median trophosome	3	Hydrothermal vent worm	[7]
Riftia pachyptila	Animalia	Annelida	central trophosome	1	Hydrothermal vent worm	[7]
Lamellibrachia luymesi	Animalia	Annelida	epidermis	3	Cold seep worm	[7]
Lamellibrachia luymesi	Animalia	Annelida	peripheral trophosome	3	Cold seep worm	[7]
Lamellibrachia luymesi	Animalia	Annelida	median trophosome	3	Cold seep worm	[7]
Lamellibrachia luymesi	Animalia	Annelida	central trophosome	3	Cold seep worm	[7]
Styela clava	Animalia	Chordata, Tunicata	epithelial cells of stomach & esophagus	420	Funnel epithelium of dorsal tubercle, lip epithelium of dorsal tubercle, mucus cells of	[8]

Species	Kingdom	group	cell type	T_c (h)	Notes	Reference
					esophagus, crest population of stomach, groove population of stomach	
Styela clava	Animalia	Chordata, Tunicata	intestinal & rectal epithelia	840	Intestinal epithelium, rectal epithelium	[8]
Styela clava	Animalia	Chordata, Tunicata	Cells of endostyle & esophagus	several months	Zone 1 of endostyle, band cells of esophagus	[8]
Mus musculus	Animalia	Chordata, mouse	duodenal crypt	10.4-13.5		Reviewed by [9]
Mus musculus	Animalia	Chordata, mouse	colon	20.9-21.8		Reviewed by [9]
Mus musculus	Animalia	Chordata, mouse	jejunal mucosa	18.75		Reviewed by [10]
Mus musculus	Animalia	Chordata, mouse	colon mucosa	16		Reviewed by [10]
Mus musculus	Animalia	Chordata, mouse	periodontal fibroblasts	33.9-42.4		Reviewed by [9]
Mus musculus	Animalia	Chordata, mouse	thymus	9.4-9.6		Reviewed by [9]
Mus musculus	Animalia	Chordata, mouse	uterine cervix	22.8		Reviewed by [9]
Mus musculus	Animalia	Chordata, mouse	erythroblasts	5.3-7.3		Reviewed by [9]
Mus musculus	Animalia	Chordata, mouse	hair follicle	13		Reviewed by [10]
Mus musculus	Animalia	Chordata, mouse	esophagus	181		Reviewed by [10]
Rattus norvegicus	Animalia	Chordata, rat	jejunal crypt	10.0-15.5		Reviewed by [9]
Rattus norvegicus	Animalia	Chordata, rat	bone	38.1		Reviewed by [9]
Rattus norvegicus	Animalia	Chordata, rat	cartilage cells	23		Reviewed by [9]
Rattus norvegicus	Animalia	Chordata, rat	tibia cartilage	54.2		Reviewed by [9]
Rattus norvegicus	Animalia	Chordata, rat	leukocytes	10.4-34.6		Reviewed by [9]
Rattus norvegicus	Animalia	Chordata, rat	erythroblasts	7.4-11.1		Reviewed by [9]
Rattus norvegicus	Animalia	Chordata, rat	thyroid follicle	120.9		Reviewed by [9]
Rattus norvegicus	Animalia	Chordata, rat	brain	15.3-23.3		Reviewed by [9]
Rattus norvegicus	Animalia	Chordata, rat	dentate gyrus	16.4		Reviewed by [9]
Rattus norvegicus	Animalia	Chordata, rat	dentate gyrus	18.7		Reviewed by [9]
Rattus norvegicus	Animalia	Chordata, rat	dentate gyrus	15.8		Reviewed by [9]
Rattus norvegicus	Animalia	Chordata, rat	tibial metaphysis	36		Reviewed by [10]
Rattus norvegicus	Animalia	Chordata, rat	tibial endosteum	57		Reviewed by [10]
Rattus norvegicus	Animalia	Chordata, rat	tibial periosteum	114		Reviewed by [10]

Species	Kingdom	group	cell type	T_c (h)	Notes	Reference
Rattus norvegicus	Animalia	Chordata, rat	condylar cartilage prechondroblast	78-114	Slows with time of year/age.	Reviewed by [11]
Rattus norvegicus	Animalia	Chordata, rat	sphenooccipital synchondrosis chondroblast	55.6		Reviewed by [11]
Rattus norvegicus	Animalia	Chordata, rat	cultured skeletoblasts	72-187		Reviewed by [11]
Rattus norvegicus	Animalia	Chordata, rat	cultured prechondroblasts	19-55	Slowed with age.	Reviewed by [11]
**Embryonic/larval animal tissue**						
Baikalospongia bacillifera	Animalia	Porifera	choanocyte in larva	13-15		[12]
Tectura scutum	Animalia	Mollusca, Gastropoda	2-4 cell stage embryo	0.87-0.55	Reared in 10°C and 14°C	[13]
Calliostoma ligatum	Animalia	Mollusca, Gastropoda	2-4 cell stage embryo	1.57-1.03	Reared in 10°C and 14°C	[13]
Littorina scutulata	Animalia	Mollusca, Gastropoda	2-4 cell stage embryo	3-1.68	Reared in 10°C and 14°C	[13]
Littorina sitkana	Animalia	Mollusca, Gastropoda	2-4 cell stage embryo	4.13-2.8	Reared in 10°C and 14°C	[13]
Lacuna vincta or variegata	Animalia	Mollusca, Gastropoda	2-4 cell stage embryo	3.85-2.6	Reared in 10°C and 14°C	[13]
Haminaea vesicula	Animalia	Mollusca, Gastropoda	2-4 cell stage embryo	3.77-1.98	Reared in 10°C and 14°C	[13]
Haminaea callidegenita	Animalia	Mollusca, Gastropoda	2-4 cell stage embryo	10.17-4.8	Reared in 10°C and 14°C	[13]
Helobdella triserialis	Animalia	Annelida, Hirudinea	embryonic cells	4-9	From Table 1, page 112	[14]
Asplanchna brightwelli	Animalia	Rotifera	Embryo (first 10 cleavages)	0.25-0.50	Lifespan ~4 days	[15]
Asplanchna brightwelli	Animalia	Rotifera	Adult/post-mitotic	0	Lifespan ~4 days	[15]
Asplanchna brightwelli	Animalia	Rotifera	Syncytial vitellarium	0	Lifespan ~4 days	[15]
Terebratalia transversa	Animalia	Brachiopoda, Articulata	2-4 cell stage embryo	1.03-0.63	Reared in 10°C and 14°C	[13]
Terebratulina unguicula	Animalia	Brachiopoda, Articulata	2-4 cell stage embryo	1.43-0.8	Reared in 10°C and 14°C	[13]
Phoronis pallida	Animalia	Phoronida	2-4 cell stage embryo	1.25-0.68	Reared in 10°C and 14°C	[13]

Species	Kingdom	group	cell type	T_c (h)	Notes	Reference
Phoronis vancouverensis	Animalia	Phoronida	2-4 cell stage embryo	2.88-2.02	Reared in 10°C and 14°C	[13]
Luidia foliolata	Animalia	Echinoderm, asteroid	2-4 cell stage embryo	1.93	Reared in 10°C	[13]
Evasterias troschelii	Animalia	Echinoderm, asteroid	2-4 cell stage embryo	1.5	Reared in 10°C	[13]
Orthasterias koehleri	Animalia	Echinoderm, asteroid	2-4 cell stage embryo	2	Reared in 10°C	[13]
Pisaster ochraceus	Animalia	Echinoderm, asteroid	2-4 cell stage embryo	1.57	Reared in 10°C	[13]
Leptasterias hexactis	Animalia	Echinoderm, asteroid	2-4 cell stage embryo	6.55	Reared in 10°C	[13]
Crossaster papposus	Animalia	Echinoderm, asteroid	2-4 cell stage embryo	2.03	Reared in 10°C	[13]
Pteraster tesselatus	Animalia	Echinoderm, asteroid	2-4 cell stage embryo	1.68	Reared in 10°C	[13]
Henricia leviuscula	Animalia	Echinoderm, asteroid	2-4 cell stage embryo	1.98	Reared in 10°C	[13]
Henricia sp. (gray armpit)	Animalia	Echinoderm, asteroid	2-4 cell stage embryo	1.73	Reared in 10°C	[13]
Henricia sp. (brooder)	Animalia	Echinoderm, asteroid	2-4 cell stage embryo	2.93	Reared in 10°C	[13]
Oikopleura dioica	Animalia	Chordata, Tunicata	2-4 cell stage embryo	0.33-0.23	Reared in 10°C and 14°C	[13]
Corella inflata	Animalia	Chordata, Tunicata	2-4 cell stage embryo	0.87-0.58	Reared in 10°C and 14°C	[13]
Ascidia paratropa	Animalia	Chordata, Tunicata	2-4 cell stage embryo	1.08-0.7	Reared in 10°C and 14°C	[13]
Boltenia villosa	Animalia	Chordata, Tunicata	2-4 cell stage embryo	0.92-0.59	Reared in 10°C and 14°C	[13]
Mus musculus	Animalia	Chordata, mouse	neural progenitor cell	8-18		[16]
Mus musculus	Animalia	Chordata, mouse	fetal neocortex (cerebral wall)	15.1		[17]
Rattus norvegicus	Animalia	Chordata, rat	retina during development	14-55		[18]
Homo sapiens	Animalia	Chordata, human	embryonic stem cell	15-16		[19]
Homo sapiens	Animalia	Chordata, human	normablast	15-18		Reviewed by [10]

Unicellular Eukaryotes

Amphidinium carteri	Protista	dinoflagellate	Whole cell	27		[20]

Species	Kingdom	group	cell type	T_c (h)	Notes	Reference
Amphidinium carteri	Protista	dinoflagellate	Light-limited	82	Cell volume also decreased with light limitation.	[20]
Amphidinium carteri	Protista	dinoflagellate	N-limited	139		[20]
Alexandrium fundyense	Protista	dinoflagellate	Whole (strain GtCA29)	60-150	Varies depending on temperature & phosphorus.	[21]
Chlamydomonas eugametos	Plantae	unicellular green algae	Whole cell	20-48	High temperatures = longer cell cycle & larger cells	[22]
Saccharomyces cerevisiae	Fungiae	yeast	Whole cell	1.65-2.37		[23]
Saccharomyces cerevisiae	Fungiae	yeast	Whole cell	2.1	Good N source (ammonia)	[24]
Saccharomyces cerevisiae	Fungiae	yeast	Whole cell	1.8	Good N source (glutamine)	[24]
Saccharomyces cerevisiae	Fungiae	yeast	Whole cell	6.7	Poor N source (proline)	[24]
Aspergillus nidulans	Fungiae	ascomycete (filamentous fungus)	Whole cell	1.5-2		[25]
Euglena gracilis	Protista	euglena	Whole cell	24-30		Reviewed by [26]
Monosiga brevicollis	Protista	Choanoflagellata	Whole cell	6		[27]
Physarum polycephalum	Fungiae	Myxomycete (slime mold)	syncytial plasmodium	8-12		Reviewed by [28]
Dictyostelium discoideum	Fungiae	slime mold	cell	9		[29]
Euplotes eurystomus	Protista	ciliated protozoan	syncytial macronuclei	14	Divides amitotically.	[30]
Euplotes eurystomus	Protista	ciliated protozoan	syncytial micronuclei	14	Divides while macronucleus is also dividing.	[30]
**Bacteria**						
Salmonella	Bacteria	bacterium	Whole cell	0.42		Reviewed by [10]
Escherichia coli	Bacteria	bacterium	Whole cell	0.67-1.5		Reviewed by [31]
Alcaligenes eutrophus	Bacteria	bacterium	Whole cell	1.267		Reviewed by [31]
Bacillus subtilis	Bacteria	bacterium	Whole cell	1.33		Reviewed by [31]

Supplemental Table 3. Estimate of percent tissue that would be shed and replaced per day for five sponge species. First the proportion of the body volume that is filled with choanocytes was estimated for the choanosome region (region that contains choanocyte chambers), then scaled up to a full sponge body with choanosome and non-choanosome regions. Annotations explain calculations and sources of the numbers used.

	Halisarca caerulea	*Spongilla lacustris*	*Sycon coactum*	*Haliclona mollis*	*Aphrocallistes vastus*
Choanocytes per field of view[a]	218[b]	177	325	177	346
Total area in a field of view (μm^2)	$3.4x10^4$ [b]	$7.5x10^4$	$7.5x10^4$	$7.5x10^4$	$2.8x10^5$
% of choanosome that is choanocytes[c]	18%[b]	7%	12%	7%	3%
Proliferation rate (% cells h^{-1})	7.24[b]	0.19	0.7	0.25	0.17
% of choanosome that is replaced (% d^{-1})[e]	31.2	0.3	2.0	0.4	0.1
% of body that is choanosome[f]	100	75	75	75	5
% of total body that is replaced (% d^{-1})	31.20	0.23	1.53	0.30	0.01

[a] For *A. vastus*, choanoblasts were considered in place of anywhere that choanocytes are mentioned.
[b] From [2]. Presented as % of total tissue that is choanocytes.
[c] Using choanocyte volume of 28 μm^3 [2] and assuming section thickness of 1 μm^3.
[d] Calculated using proliferation rates measured as % cells h^{-1}.
[e] Calculated by multiplying the proliferation rate by the percent of the choanosome that is choanocytes
[f] 75% was assumed for all species based on relative component of the choanosome compared to other regions of the tissue (e.g. pinacoderm or cortex) except for *H. caerulea* (noted above in footnote b: 18% of the total body was choanocytes) and *A. vastus*. The % of body that is choanosome for *Aphrocallistes vastus* was estimated to be much less because only the growing edge at the tip of the sponge has proliferation occurring.
[g] Calculated by multiplying the % of the choanosome replaced by the overall body that is choanosome.

Supplemental Table 4. Comparison of carbon that could be lost to cell replacement and the proportion of the total carbon budget of the sponge that cell replacement makes up. Annotations explain calculations and sources of the numbers used.

	Halisarca caerulea	*Haliclona mollis*	*Aphrocallistes vastus*
Carbon lost to cell replacement			
Cells per chamber		139[a]	87[b]
Proliferation rate (% h^{-1})	7.24[f]	0.25	0.17
Choanocytes lost per day (cells d^{-1})[c]		8.3	3.5
Carbon used per chamber per day (pg C $chamber^{-1}$ d^{-1})[d]		23.4	9.9
Density of chambers (chambers ml^{-1})		$2.68x10^{6}$ [a]	$1.88x10^{6}$ [b]
Choanocytes replaced in 1 ml of sponge per day (cells ml $sponge^{-1}$ d^{-1})[e]		2 x 10^{7}	7 x 10^{6}
Carbon spent in 1-ml sponge per day from cell turnover (µmol C ml $sponge^{-1}$ h^{-1})	13.80[f]	0.22	0.06
Carbon consumed through feeding			
Ambient picoplankton concentration (cells ml^{-1})		$7.57x10^{5}$ [g]	$6.86x10^{5}$ [h]
Removal efficiency (%)		88%[g]	78.6%[h]
Volumetric flow rate (ml ml $sponge^{-1}$ min^{-1})		48.6[a]	21[i]
Grazing rate (α, ml water cleared ml $sponge^{-1}$ min^{-1})[j]		42.8	16.4
Bacteria consumed (cells d^{-1})[k]		4.66 x 10^{10}	1.62 x 10^{10}
Carbon consumed (µmol C ml $sponge^{-1}$ h^{-1})	18.5[l]	4.89	1.70
% of total carbon consumed that is spent on cell replacement (% d^{-1})	75%	4%	3.80%

[a] From [32]
[b] From [33], as collars per chamber. Several enucleate collars branch from a choanoblast, so 260 chambers was divided by 3 collars/choanoblast.
[c] Calculated by multiplying the proliferation rate by the average number of cells in a chamber.
[d] Calculated assuming 2.8 pg C $choanocyte^{-1}$ [2]
[e] Calculated by multiplying the choanocytes replaced per day by the chamber density
[f] From [2]
[g] From [34]
[h] From [35]
[i] Calculated using the volumetric flow rate per osculum (0.000106 m^3 $osculum^{-1}$ s^{-1}, [35]) and the average volume of an individual of *A. vastus* (304.43 ml, n=7)
[j] Calculated by multiplying the removal efficiency by the volumetric flow rate.
[k] Calculated assuming 30.2 fg C $cell^{-1}$ for bacteria (picoplankton) [36]
[l] From [37]. *H. caerulea* is the only one of the three species listed here that takes in the majority of its carbon as DOC; the other two species did not take up measurable amounts of DOC [34, 35] so only bacterioplankton feeding was considered for them when calculating the carbon consumed.

Supplemental Video 1. Time-lapse video of a choanocyte chamber being moved by a cell from the mesohyl. The choanocyte chamber (outlined in orange initially) was held by a cell from the mesohyl that attached to it with two pseudopodia (outlined in green initially). The video corresponds with the still image shown in Supplemental Figure 3.

Supplemental Video 2. Time-lapse video of cells from the mesohyl that immigrated into choanocyte chambers. Two cells, pseudocoloured red and green, are seen moving through the mesohyl and migrating into two different choanocyte chambers. The red cell in the video corresponds with the still images shown in Figure 2.

Supplemental methods

Husbandry and experimental setup

Freshwater sponges were kept in 18°C lake water, refreshed daily. Marine sponges were maintained in flow-through seawater tables (9-10°C) at BMSC or, for the *A. vastus* collected by SCUBA, fragments 1 cm^2 were kept in 0.5 L containers of seawater at 9°C in an incubator at the Marine Technology Centre, University of Victoria, in Sidney, B.C.

Proliferation rates in *S. lacustris* hatched from gemmules were compared to those of adult sponges. Gemmules were collected from Rosseau Lake, B.C. by SPL in December 2011, kept at 4°C in unfiltered lake water aerated monthly. Gemmules were hatched and cultured in April 2012 as described by Elliott and Leys [38]. Once a full aquiferous system had developed 5 d post-hatching (dph), sponges were incubated in 50 µM EdU in M-medium [39] for between 4 and 72 h.

References for Supplemental Materials

1. Shore R.E. 1971 Growth and renewal studies of the choanocyte population in *Hymeniacidon sinapium* (Porifera: Demospongiae) using Colcemid and 3-H thymidine. *J Exp Biol* **177**, 359-364.
2. De Goeij J.M., De Kluijver A., Van Duyl F.C., Vacelet J., Wijffels R.H., De Goeij A.F.P.M., Cleutjens J.P.M., Schutte B. 2009 Cell kinetics of the marine sponge *Halisarca caerulea* reveal rapid cell turnover and shedding. *J Exp Biol* **212**, 3892-3900. (doi:10.1242/jeb.034561).
3. Bosch T.C., David C.N. 1984 Growth regulation in *Hydra*: relationship between epithelial cell cycle length and growth rate. *Dev Biol* **104**(1), 161-171.
4. Holstein T.W., David C.N. 1990 Cell cycle length, cell size, and proliferation rate in *Hydra* stem cells. *Dev Biol* **142**, 392-400.
5. Martínez-Expósito M.J., Pasantes J.J., Méndez J. 1994 Proliferation kinetics of mussel (*Mytilus galloprovincialis*) gill cells. *Mar Biol* **120**(1), 41-45. (doi:10.1007/BF00381940).
6. Zaldibar B., Cancio I., Marigómez I. 2004 Circatidal variation in epithelial cell proliferation in the mussel digestive gland and stomach. *Cell Tissue Res* **318**(2), 395-402. (doi:10.1007/s00441-004-0960-0).
7. Pflugfelder B., Cary S.C., Bright M. 2009 Dynamics of cell proliferation and apoptosis reflect different life strategies in hydrothermal vent and cold seep vestimentiferan tubeworms. *Cell Tissue Res* **337**(1), 149-165. (doi:10.1007/s00441-009-0811-0).
8. Ermak T.H. 1975 Cell proliferation in the digestive tract of *Styela clava* (Urochordata: Ascidiacea) as revealed by autoradiography with tritiated thymidine. *J Exp Zool* **194**(3), 449-465. (doi:10.1002/jez.1401940302).
9. Guiguet M., Kupiec J.-J., Valleron J. 1984 A systematic study of the variability of cell cycle phase durations in experimental mammalian systems. In *Cell Cycle Clocks* (ed. Edmunds Jr. L.N.), pp. 97-112. New York, Marcel Dekker, Inc.
10. Van't Hof J. 1965 Relationships between mitotic cycle duration, S period duration and the average rate of DNA synthesis in the root meristem cells of several plants. *Exp Cell Res* **39**(1), 48-58. (doi:http://dx.doi.org/10.1016/0014-4827(65)90006-6).
11. Petrovic A.G., Oudet C.L., Stutzmann J.J. 1984 Temporal organization of rat and human skeletal cells: Circadian frequency and quantization of cell generation time. In *Cell Cycle Clocks* (ed. Edmunds Jr. L.N.), pp. 325-350. New York, Marcel Dekker, Inc.
12. Efremova S.M., Efremov V.I. 1977 Proliferation cellulaire chez la larve nageante de l'éponge d'eau douce: *Baikalospongia bacillifera* (Dybowski). *Colloques Internationaux du C N R S* **291**, 59-65.
13. Strathmann R.R., Staver J.M., Hoffman J.R. 2002 Risk and the evolution of cell-cycle durations of embryos. *Evolution* **56**(4), 708-720. (doi:10.1111/j.0014-3820.2002.tb01382.x).
14. Bissen S.T., Weisblat D.A. 1989 The durations and compositions of cell cycles in leech, *Helobdella triserialis*. *Development* **106**(1), 105-118.
15. Birky C.W., Bignami R.Z., Bentfeld M.J. 1967 Nuclear and cytoplasmic DNA synthesis in adult and embryonic rotifers. *Biol Bull* **133**(3), 502-509. (doi:10.2307/1539913).

16. Al-Kofahi O., Radke R.J., Goderie S.K., Shen Q., Temple S., Roysam B. 2006 Automated cell lineage construction: a rapid method to analyze clonal development with murine neural progenitor cells. *Cell Cycle* **5**(3), 327-335.
17. Takahashi T., Nowakowski R., Caviness V. 1993 Cell cycle parameters and patterns of nuclear movement in the neocortical proliferative zone of the fetal mouse. *The Journal of Neuroscience* **13**(2), 820-833.
18. Alexiades M.R., Cepko C. 1996 Quantitative analysis of proliferation and cell cycle length during development of the rat retina. *Dev Dyn* **205**(3), 293-307. (doi:10.1002/(SICI)1097-0177(199603)205:3<293::AID-AJA9>3.0.CO;2-D).
19. Becker K.A., Ghule P.N., Therrien J.A., Lian J.B., Stein J.L., van Wijnen A.J., Stein G.S. 2006 Self-renewal of human embryonic stem cells is supported by a shortened G1 cell cycle phase. *J Cell Physiol* **209**(3), 883-893. (doi:10.1002/jcp.20776).
20. Olson R.J., Chisholm S.W. 1986 Effects of light and nitrogen limitation on the cell cycle of the dinoflagellate *Amphidinium carteri*. *J Plankton Res* **8**(4), 785-793. (doi:10.1093/plankt/8.4.785).
21. Taroncher-Oldenburg G., Kulis D.M., Anderson D.M. 1999 Coupling of saxitoxin biosynthesis to the G1 phase of the cell cycle in the dinoflagellate *Alexandrin fundyense*: temperature and nutrient effects. *Nat Toxins* **7**(5), 207-219. (doi:10.1002/1522-7189(200009/10)7:5<207::AID-NT61>3.0.CO;2-Q).
22. Zachleder V., Van Den Ende H. 1992 Cell cycle events in the green alga *Chlamydomonas eugametos* and their control by environmental factors. *J Cell Sci* **102**(3), 469-474.
23. Brewer B.J., Chlebowicz-Sledziewska E., L F.W. 1984 Cell cycle phases in the unequal mother/daughter cell cycles of *Saccharomyces cerevisiae*. *Mol Cell Biol* **4**(11), 2529-2531.
24. Rivin C.J., Fangman W.L. 1980 Cell cycle phase expansion in nitrogen-limited cultures of *Saccharomyces cerevisiae*. *The Journal of Cell Biology* **85**(1), 96-107. (doi:10.1083/jcb.85.1.96).
25. Bergen L.G., Morris N.R. 1983 Kinetics of the nuclear division cycle of *Aspergillus nidulans*. *J Bacteriol* **156**(1), 155-160.
26. Adams K.J., Weiler C.S., Edmunds Jr. L.N. 1984 Photoperiodic control of cell division in *Euglena* and *Ceratium*. In *Cell Cycle Clocks* (ed. Edmunds Jr. L.N.), pp. 395-430. New York, Marcel Dekker, Inc.
27. King N., Hittinger C.T., Carroll S.B. 2003 Evolution of key cell signaling and adhesion protein families predates animal origins. *Science* **301**(5631), 361-363. (doi:10.1126/science.1083853).
28. Tyson J.J., Sachsenmaier W. 1984 The control of nuclear division in *Physarum polycephalum*. In *Cell Cycle Clocks* (ed. Edmunds Jr. L.N.), pp. 253-270. New York, Marcel Dekker, Inc.
29. McDonald S.A., Durston A.J. 1984 The cell cycle and sorting behaviour in *Dictyostelium discoideum*. *J Cell Sci* **66**(1), 195-204.
30. Prescott D.M., Kimball R.F., Carrier R.F. 1962 Comparison between the timing of micronuclear and macronuclear DNA synthesis in *Euplotes eurystomus*. *The Journal of Cell Biology* **13**(1), 175-176. (doi:10.1083/jcb.13.1.175).
31. Poole R.K. 1984 Is energy metabolism in the prokaryotic cell cycle manifestly coupled to a clock? In *Cell Cycle Clocks* (ed. Edmunds Jr. L.N.), pp. 193-208. New York, Marcel Dekker, Inc.

32. Ludeman D.A. 2015 Sponges as sensitive animals: sensory systems and energetics of filtration in demosponges [MSc thesis]. Edmonton, AB, University of Alberta.
33. Leys S.P., Yahel G., Reidenbach M.A., Tunnicliffe V., Shavit U., Reiswig H.M. 2011 The sponge pump: the role of current induced flow in the design of the sponge body plan. *PLoS ONE* **6**(12), e27787. (doi:10.1371/journal.pone.0027787).
34. Jimenez Tejero E. 2011 Nutrient fluxes in marine sponges: methodology, geographical variability and the role of associated microorganisms [PhD thesis], Universidad Politécnica de Cataluña.
35. Kahn A.S., Yahel G., Chu J.W.F., Tunnicliffe V., Leys S.P. 2015 Benthic grazing and carbon sequestration by deep-water glass sponge reefs. *Limnol Oceanogr* **60**(1), 78-88. (doi:10.1002/lno.10002).
36. Fukuda R., Ogawa H., Nagata T., Koike I. 1998 Direct determination of carbon and nitrogen contents of natural bacterial assemblages in marine environments. *Appl Environ Microbiol* **64**(9), 3352-3358.
37. De Goeij J.M., Van Den Berg H., van Oostveen M.M., Epping E.E.H.G., van Duyl F.C.F. 2008 Major bulk dissolved organic carbon (DOC) removal by encrusting coral reef cavity sponges. *Mar Ecol Prog Ser* **357**, 139-151. (doi:10.3354/meps07403).
38. Elliott G.R.D., Leys S.P. 2007 Coordinated contractions effectively expel water from the aquiferous system of a freshwater sponge. *J Exp Biol* **210**, 3736-3748. (doi:10.1242/jeb.003392).
39. Rasmont R. 1961 Une technique de culture des éponges d'eau douce en milieu contrôlé. *Ann Soc R Zool Belg* **91**, 149-155.
